# Decision making in patients with acute abdominal pain at a university and at a rural hospital: does the value of abdominal sonography differ?

**DOI:** 10.1186/1749-7922-3-29

**Published:** 2008-10-08

**Authors:** Aristomenis K Exadaktylos, Charlotte Sadowski-Cron, Paul Mäder, Monika Weissmann, Hans Peter Dinkel, Marco Negri, Heinz Zimmermann

**Affiliations:** 1 Department of Emergency Medicine, University of Berne, Inselspital, Berne, Switzerland; 2 Rural Hospital Frutigen, Switzerland; 3 Department of Radiology University of Berne, Inselspital, Berne, Switzerland

## Abstract

**Introduction and objectives:**

Abdominal sonography is regarded as a quick and effective diagnostic tool for acute abdominal pain in emergency medicine. However, final diagnosis is usually based on a combination of various clinical examinations and radiography. The role of sonography in the decision making process at a hospital with advanced imaging capabilities versus a hospital with limited imaging capabilities but more experienced clinicians is unclear.

The aim of this pilot study was to assess the relative importance of sonography and its influence on the clinical management of acute abdominal pain, at two Swiss hospitals, a university hospital (UH) and a rural hospital (RH).

**Methods:**

161 patients were prospectively examined clinically. Blood tests and sonography were performed in all patients. Patients younger than 18 years and patients with trauma were excluded. In both hospitals, the diagnosis before and after ultrasonography was registered in a protocol. Certainty of the diagnosis was expressed on a scale from 0% to 100%.

The decision processes used to manage patients before and after they underwent sonography were compared. The diagnosis at discharge was compared to the diagnosis 2 – 6 weeks thereafter.

**Results:**

Sensitivity, specificity and accuracy of sonography were high: 94%, 88% and 91%, respectively.

At the UH, management after sonography changed in only 14% of cases, compared to 27% at the RH. Additional tests were more frequently added at the UH (30%) than at the RH (18%), but had no influence on the decision making process-whether to operate or not. At the UH, the diagnosis was missed in one (1%) patient, but in three (5%) patients at the RH. No significant difference was found between the two hospitals in frequency of management changes due to sonography or in the correctness of the diagnosis.

**Conclusion:**

Knowing that sonography has high sensitivity, specificity and accuracy in the diagnosis of acute abdominal pain, one would assume it would be an important diagnostic tool, particularly at the RH, where tests/imaging studies are rare.

However, our pilot study indicates that sonography provides important diagnostic information in only a minority of patients with acute abdominal pain.

Sonography was more important at the rural hospital than at the university hospital. Further costly examinations are generally ordered for verification, but these additional tests change the final treatment plan in very few patients.

## Introduction

Acute abdominal pain is a non-specific symptom of many diseases. An efficient initial diagnostic evaluation, including physical examination and blood tests, is performed in most cases [1,2]. Different authors have shown that sonography adds up to 40% more information to these clinical examinations [3-6] and leads to a change in management in 20% of cases [7-9]. Thus, sonography is considered an important diagnostic tool in emergency departments (ED) [9-11].

The accuracy of sonography has been found to be between 71–98% for acute appendicitis and billiary tract disease [4,8,11]. The sensitivity and specificity of sonography are high in the diagnosis of cholecystitis, ileus and diverticulitis [3,5,12] but rather low in the diagnosis of appendicitis [13]. The accuracy of CT is superior to that of sonography in the diagnosis of acute appendicitis [9,14-16], but a CT-scan is rarely available at rural hospitals (RH) in Switzerland. However, CT scans [17,18], diagnostic laparoscopy [18], clinical scores [4,8,19,20] and the use of diagnostic algorithms [4,21] have been shown to be helpful in the decision making process. In many unclear situations, prolonged observation is used too, but is a financial burden, as it contributes to increasing hospital costs [3,22,23]. It is obvious that the decision making process in patients with acute abdominal pain is still a major challenge, as in up to 45% of cases no specific diagnosis is made [7,10,24]. The aim of this pilot study was therefore to further clarify to what extent sonography influences the decision making process in patients with acute abdominal pain at a university hospital (UH), compared with a rural hospital (RH), and also addressed the issue of how additional examinations influence surgical management.

To the best of our knowledge, this is the first study comparing the impact of sonography at a university and rural hospital emergency unit under everyday situations.

## Materials and methods

This investigation was a prospective study of a convenience sample of 161 consecutive patients with acute abdominal pain who were treated in the emergency department of both hospitals, 106 at the University Hospital Inselspital in Berne (UH) and 55 at a small rural hospital (RH), both in the district of the Bernese Alps, Switzerland. Patients younger than 18 years and trauma patients were excluded.

At both hospitals, only patients were included in the study when they had abdominal symptoms of unclear origin. Patients with clinically clear findings were not given an unnecessary sonography and the usual procedure was not changed. The UH has approximately 1000 beds, serving 1.5 million people in the region. Over 50,000 in-patients and 150,000 out-patients are treated annually, including 30,000 in the adult emergency department. Sonography, CT, MRI, gastroscopy and colonoscopy and laboratory tests are available for the diagnosis of the acute abdomen 24 hours a day. The RH has approximately 50 beds serving 20,000 inhabitants and several thousand tourists per year. Over 2,000 in-patients and 6,000 out-patients are treated annually. Sonography, X-Ray, gastroscopy and colonoscopy and laboratory examinations are available 24 hours a day. For advanced diagnostic procedures, patients have to be transported to the next district hospital, which is 25–50 km away.

**Figure 1:**
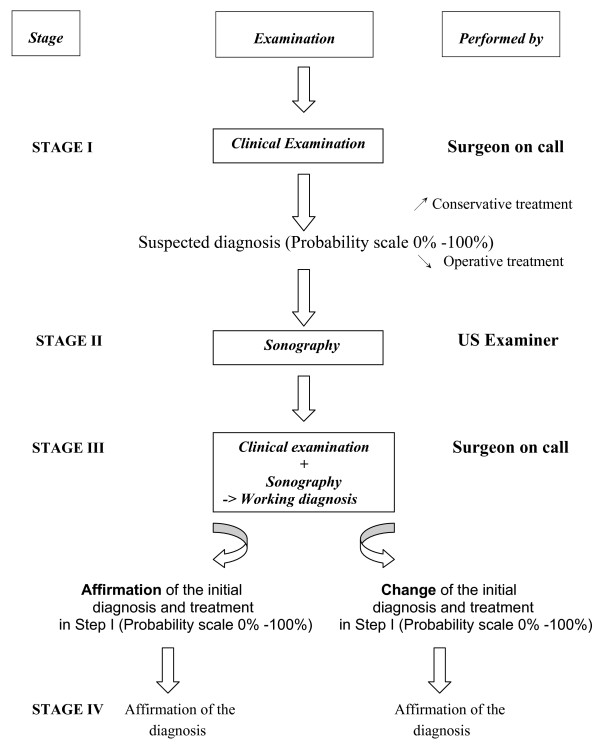
The diagnostic pathway.

At both the UH and the RH, as illustrated in Fig. 1, the diagnosis before and after sonography was registered by the surgeon on call at stage I and III and by the registrar who performed the examination at stage II. The stages are defined below.

The probability of the diagnosis at each of these stages was expressed on a scale from 0% to 100% (open in scale), broken down into the following categories.

0 to 10%: unlikely

≥ 10% to ≤ 60%: low to intermediate probability

≥ 60% to ≤ 90%: probable

≥ 90%: definitive diagnosis

The surgeon on call at the UH performed and interpreted the clinical examinations and defined the management plan. At the RH, the chief physician of the emergency department was involved in all cases.

At the UH, an experienced resident or fellow in radiology and at the RH a senior hospital physician performed and interpreted the ultrasound examinations. All doctors performing the ultrasound examination had a formal and accredited training therein. The ultrasound studies were documented at both hospitals as still images and reviewed by senior radiologists.

An Acuson 128XPIO with a 3.5 MHz transducer was used at the UH, and an Acuson 74XER with a 5 MHZ transducer at the RH. Curvilinear transducers and linear arrays were used, but no endocavitary probe True positive and true negative examinations, were recorded, together with sensitivity, specificity and accuracy.

The diagnoses were classified as appendicitis, cholecystitis, gynaecological disease, nephrolithiasis or other diseases. The surgeon had to allocate his diagnosis to one of these five groups (see Table 1 and Table 2).

**Table 1:** Frequencies of Diagnoses at Stages D 1–4 of the Decision-Making Process % (n)

**UH (n = 106)** **RH (n = 55)**	**Stage I** Clinical diagnosis	**Stage II** diagnosis by sonography	**Stage III** Working diagnosis	**Stage IV** final diagnosis after 2 weeks
**Appendicitis**				
**UH**	47% (50)	18% (19)	23% (24)	22% (23)
**RH**	33% (18)	24% (13)	24% (13)	22% (12)

**Cholecystitis**				
**UH**	22% (23)	10% (11)	10% (11)	10% (11)
**RH**	11% (6)	5% (3)	5% (3)	5% (3)

**Gynaecologica disease**				
**UH**	11% (12)	9% (10)	11% (12)	9% (10)
**RH**	13% (7)	7% (4)	7% (4)	7% (4)

**Nephrolithiasis**				
**UH**	12% (13)	8% (8)	11% (12)	11% (12)
**RH**	9% (5)	9% (5)	9% (5)	9% (5)

**Other disease**				
**UH**	8% (8)	55% (58)	44% (47)	44% (47)**
**RH**	35% (19)	55% (30)	55% (30)	56% (31)

** The 47 (31) final diagnoses at the UH (RH) were:

Constipation 14 (0), gastroenteritis 13 (8), NSAP 5 (5), diverticulitis 4 (2), gastritis 3 (0), UTI 2 (0), spleen infarction 1 (0), musculoskeletal pain 1 (2), ovulation pain 1 (0), diverticulosis 1 (0), incarcerated hernia 1 (0), pancreatitis 1 (1), ileus 0 (2), coecal necrosis 0 (1), Meckel 's diverticulum 0 (1), haematoma of the abdominal wall 0 (1), bladder retention 0 (1), coecal volvolus 0 (1), colitis 0 (1), hepatocellular carcinoma 0 (1), intraabdominal tumour of unknown origin 0 (1), lymphoma 0 (1), colonic cancer 0 (1), pyelonephritis 0 (1)

**Table 2:** Allocation of the Individual Diagnoses at Each Stage n (%)

***Diagnosis*** **UH (n = 106)** **RH (n = 55)**	***Stage I*** *(Basic clinical examination)*	***Stage II*** *(Sonography)*			***Stage III*** *(Working diagnosis)*	***Stage IV*** *(Histo; Operating surgeon report; Clinical process)*
		**1**	**2**	3(*)		
**Appendicitis**						
**UH**	50 (47%)	19 (18%)	0 (0%)	31 (29%)°	24 (23%)A	23 (22%)
**RH**	18 (33%)	11 (20%)	2 (4%)	7 (13%)°	13(24%)	12 (22%)

**Cholecystitis**						
**UH**	23 (22%)	11 (10%)	0 (0%)	12 (11%)^1^	11 (10%)	11 (10%)
**RH**	6 (11%)	2 (4%)	1 (2%)	4 (7%)^1^	3 (5%)	3 (5%)

**Gyn. Disease**						
**UH**	12 (11%)	8 (8%)	2 (2%)	2 (2%) ^2^	10 (9%)	10 (9%)
**RH**	7(13%)	3 (5%)	1 (2%)	4 (7%) ^2^	4 (7%)	4 (7%)

**Nephrolithiasis**						
**UH**	13 (12%)	8 (8%)	0 (0%)	5 (5%)^3^	12 (11%) B	12 (11%)
**RH**	5 (9%)	3 (5%)	2 (4%)	3 (5%)^3^	5 (9%)	5 (9%)

(*) **1**: affirmed with sonography

**2**: newly diagnosed by sonography (initially an other diagnosis at Stage I)

**3**: excluded with sonography

° 10 × Constipation/15 × Gastroenteritis/2 × NSAP/4 × Diverticulitis (UH)

1 × Mesenterialinfarct/3 × Gastroenteritis/2 × NSAP/1 × Diverticulitis (RH)

^1^6 × Gastroenteritis/1 × Musculoskeletal/4 × Constipation/1 × Spleen infarction (UH)

2 × NSAP/1 × Musculoskeletal/1 × Liver Cancer (RH)

^2^2 × Urinary tract infection

1 × Adhesive ileus/1 × Coecumvolvulus/1 × Nephrolithiasis/1 × Appendicitis perf (RH)

^3^1 × Pyelonephritis/3 × Condition after nephrolith loss, diagnosis with CT/1 × praevesical Concrement missed by sonography (UH)

1 × Gastritis/1 x NSAP/1 × Musculoskeletal (RH)

A 5 × the diagnosis was made by the surgeon on call despite negative ultrasonography, in some cases after additional tests (eg blood tests)

B 4 × the diagnosis was made by the surgeon on call despite negative ultrasonography, in some cases after additional tests (eg blood tests)

### Stage I

The diagnostic pathway [Fig. 1] was as follows: After basic clinical examination (history taking, clinical examination and blood test), the surgeon on call recorded the probability of the diagnosis in the protocol and decided whether the patient should be treated by operation or conservatively.

Blood count, leukocytes, CRP, liver function tests, amylase, creatinine and urine dipstick were performed.

### Stage II

[Fig. 1]: Sonography was performed and interpreted as mentioned above. No examiner had access to history, physical examination or lab results.

### Stage III

[Fig. 1]: Knowing the result of the sonography, the surgeon on call had to decide which steps to take next on the basis of clinical necessity. Options included surgery, conservative treatment, discharge, or additional examinations, such as CT-scan, endoscopic retrograde cholangiopancreatography (ERCP), gastroscopy, and others. Women whose pain was suspected to have gynaecological causes following the investigations mentioned above were seen at both hospitals by the gynaecologist on call (UH 6%/RH 9%).

### Stage IV

[Fig. 1]: The final diagnosis was either obtained from the operating surgeon's report or in a telephone interview with the family physician 2 – 4 weeks after presentation to the ED.

The term *non-specific abdominal pain *(NSAP) was used when no diagnosis was found.

We defined and identified the 3 following outcomes (table 2/table 3):

**Table 3:** Change of Management Plan after Sonography

	**Conservative -> Operative**		**Operative -> Conservative**		**Additional Examinations**		**Change kind of operation**		**Other (Transfer to UH)**		**Total**
	
	**14 × (13%)**		**1× (1%)**		**30× (28%) ①**		**0**		**0**		**15 ×**
	
**UH**	**Stage I**	**Stage III**	**Stage I**	**Stage III**	**Stage I**	**Stage III**	**Stage I**	**Stage III**	**Stage I**	**Stage III**	
	
	Pelvic inflamatory disease 2× Cholec. 8× App. Ⓑ 2× App. Ⓑ Gyn.	Abscess CCE AE Process = AE EUG	Append.	Enteritis?Ⓐ	Table 4	Table 4					
**RH**	**4 × (7%)**		**3 × (6%)**		**2 × (4%)**		**3 × (6%)**		**3 × (6%)**		**15 ×**
	
	Enteritis Gastritis Adnex. Append.	Append. Cholecy. Adnex. ^1^ Append.^2^	Adnex. Append. Append.	Nephroli. Gastroen. Diverticulitis.	Pancreat. Diverticulitis.	Pancreat.^3^ Meckel °	Ovar cys. Diverticulitis. Adnexitis	Ileus Adnexitis App. perf.	Append. Ovar cys. Gastritis	Mes. Inf. Volvulu Lymphom	

① The additional examinations did not contribute to a change from initial management in any case. The additional examinations were ordered independent of the results of the sonography.

Ⓐ In the clinical process diagnosis of appendicitis and secondary operation.

Ⓑ After basic clinical examination unlikely or low probability of appendicitis.

^1 ^Basic clinical examination and sonography unclear.

^2 ^Basic clinical examination and sonography unclear.

^3 ^ERCP after sonography

° CT after sonography

+ 5× Change of the conservative therapy at RH after sonography:

Nephrolithiasis -> Refluxoesophagitis

Hernia -> Nephrolithiasis

Cholecystolithiasis -> Musculoskeletal

Nephrolithiasis -> Musculoskeletal

Cholecystolithiasis -> Liver tumour

EUG = Extra uterine gravity; AE = Appendectomy; CCE = Cholecystectomy

1. The number of times the clinical management was changed due to the sonography (Stage I -> Stage III)

2. The number of times the diagnosis by sonography was correct (Stage II -> Stage IV)

3. The differences between UH and RH

### Statistics

All information was entered in a computerised database by means of a standard PC-based spread sheet and statistics were calculated with the assistance of a biostatistician. Patient groups were compared using the Mann-Whitney U-test, since criteria for normal distribution were not fulfilled. Categorical data were analysed by the Chi-square test, or the Fisher exact test if the expected samples size was smaller than five. A p-value smaller than 0.05 was considered significant.

## Results

At the UH, there were 68 (72%) male patients and 48 (28%) female patients with a median age of 35 years (range 18–88 years). At the RH, there were 22 (40%) male patients and 33 (60%) female patients, with a median age of 40 years (range 18–87 years). The time that elapsed from the first until the final clinical evaluation ranged between 30 minutes and 2 hours at both hospitals.

The difference in male: female ratio at the two hospitals is most likely due to the fact that at the RH every patient is seen in the emergency department, whereas at the UH females with acute abdominal pain are also treated by the emergency department of gynaecology, which was not involved in this study.

Although there were more female patients seen at the RH, the number of gynaecological diagnoses found was in the same range at the two hospitals (UH 12%/RH 7%).

The management changed in response to the sonography results in 14% (15/106) of patients at the UH but in 27% (15/55) of patients at the RH [Table 3]. Specificity and accuracy of sonography were higher at the RH than at the UH: 94% vs. 82%, respectively [Table 4]. At the UH, there were10 false negative and 3 false positive diagnoses. However, they did not influence the decision making process, perhaps because the surgeon ignored them or did not believe them; at the RH, there were 2 false positive and 1 false negative diagnoses.

**Table 4:** Sensitivity, Specificity and Accuracy of Sonography at the UH and RH

	**True Positive**	**Sensitivity**	**True Negative**	**Specificity**	**Total**	**False positive**	**False negative**	**Total**	**Accuracy**
**UH**	50	93%	43	82%	93	3	10	13	87%

**RH**	29	95%	23	94%	52	2	1	3	95%

**Total**	79	94%	66	88%	145	5	11	16	91%

True positive and negative findings where the sonographic diagnosis corresponded to the intraoperative finding or to the final diagnosis. False positives or negatives were sonographic findings that were not identical to the diagnosis after 2 weeks.

Accuracy was defined as the rate of correct final diagnosis, reconfirmed after 2 weeks.

Sonography was helpful in discarding the initial diagnosis in 29% of cases and in affirming it in 32%. In the remaining 39% of cases, sonography did not make any contribution to the diagnosis of acute abdominal pain.

In one third to one half of the patients who entered the emergency room with acute abdominal pain, appendicitis was suspected after clinical examination [Stage I, Table 1]. However, appendicitis could be confirmed by ultrasonic examination in only half of the cases [Stage II, Table 1 and 2]. For cholecystitis, nephrolithiasis and gynaecological disease, ultrasonic examination rarely changed the clinical diagnosis [Stage I -> Stage IV; Table 1 and 2].

Additional examinations were more often requested at the UH (30%; 32 patients) than at the RH (18%; 10 patients) and led to the final diagnosis at the UH in all but 1 (1%) patient (missed benign kidney tumor). The additional examinations at the UH were as follows: CT scan (20), intravenous pyelography (1), colon enema (1), endoscopic retrograde cholangiopancreaticography (2), gynaecological examination (6), diagnostic laparoscopy (0), gastroscopy (2). The additional examinations at the RH were as follows: CT scan (2), intravenous pyelography (1), colon enema (0), endoscopic retrograde cholangiopancreaticography (2), gynaecological examination (1), diagnostic laparoscopy (4), gastroscopy (0). At the RH, one diagnosis of appendicitis was missed by sonography and the patient was operated on 2 weeks later. One suspected appendicitis turned out to be gastroenteritis, and one Meckel's diverticulum was misdiagnosed as sigmoid diverticulitis. NSAP was diagnosed at discharge in 5% (5/106) of the patients at the UH and 9% (5/55) at the RH. The difference between the two hospitals was not significant (p > 0.05).

## Discussion

Abdominal ultrasonography of patients with acute abdominal pain is very helpful for the confirmation or exclusion of clinically suspected appendicitis, billiary tract disease and aortic aneurysm and thus is an important diagnostic tool, albeit in a minority of patients [Table 3].

As a result of sonographic findings, the surgeon on call changed his initial decision as to whether to operate or to observe in 14% (15/106) of the patients at the university hospital, but in 27% (15/55) of the patients at the rural hospital [Table 3]. However, the difference was not statistically significant (p > 0.5), perhaps because of the sample size. Further, the accuracy of sonography was higher at the RH than at the UH [Table 4]. The surgeon on call might therefore have been aware of the limitation of the sonography at the UH and have chosen not to rely on the investigation without additional examinations. However, there were 3 cases (5%) misdiagnosed at the RH [Table 4].

The differences in the accuracy of sonography between the RH and the UH in our study can be explained by the fact that sonography is operator dependent and requires dedication and experience: at the RH an experienced physician performed all examinations, while at the UH the majority of ultrasounds were performed by ER residents or radiology fellows with different levels of experience. Thus sonography has great weight in decision making related to the diagnosis of acute abdominal pain at the RH. At the RH, the diagnostic possibilities are limited but the attending surgeon could, due to his great clinical experience and the long time collaboration with the sonographer, place more trust in the results of the sonography.

Our average accuracy rate of 91% in the diagnosis of acute abdominal pain is in accordance with most authors [12,15,16,25,26]. However, 10 false negative sonographies at the UH illustrate the limits of sonographers. The examiners' varied levels of experience is evident and has implications for using diagnostic sonography as mentioned above. Most authors claim that sonography increases costs without improving diagnostic performance [9,27,28]. In fact, at the UH, 32 (30%) other examinations in addition to sonography were thought to be necessary to reach a final diagnosis. Nevertheless, our study found that these costly examinations had no influence on the final diagnosis either. Further studies are urgently needed to clarify how much expensive additional examinations contribute to decision making in patients with acute abdominal pain.

The rate of non-specific abdominal pain (NSAP) at discharge was 5% (5/106) at the UH and 9% (5/55) at the RH, and no causes of abdominal pain were found in these patients 2 weeks later. These rates of NSAP are low compared to the frequencies of 25–40% reported from other centres [9,10,29].

It is often proposed that sonography should be used because of its reliability and simplicity. It has also been claimed that the correct diagnosis can be obtained after a short training period – even by non-radiologists [30]. Our results call such recommendations into doubt.

## Limitations

This study has limitations. Firstly, our study population is limited and the patient distributions (age, gender) among central hospital and urban hospital were not matched. This might influence the results, as some pathologies may change with age and differ between men and women. Secondly, there were differences between the two hospitals with respect to the experience of the sonographer and the availability of additional tests. There may be also be a bias in patient selection; some patients were excluded only on the basis of the surgeon's decision.

Due to these and other differences between the two hospitals, no general recommendations can be made on the basis of the data from this study. Larger studies are necessary, which should consider the special conditions of the individual hospitals and include economic aspects.

## Conclusion

Knowing that sonography has high sensitivity, specificity and accuracy in the diagnosis of acute abdominal pain, one would assume it would be an important diagnostic tool, particularly at the RH, where tests/imaging studies are rare.

However, our pilot study indicates that sonography provides important diagnostic information in only a minority of patients with acute abdominal pain.

Sonography was more important at the rural hospital than at the university hospital

In a time when medical expenses are rising steeply, further studies are urgently needed to investigate to what extent expensive additional examinations contribute to the decision making plan in patients with acute abdominal pain.

This study can serve as a pilot for future well designed and methodologically stringent studies

## Competing interests

The authors declare that they have no competing interests.

## Authors' contributions

AKE wrote and revised paper. CSC organised study and planned study design. PM planned study design. MW recruited patients and collected patient data. HPD supervised performed and interpreted sonographies at UH. MN supervised performed and interpreted sonographies at RH. HZ designed study.
